# The complete mitochondrial genome of the stalk-forming diatom *Didymosphenia Geminata*

**DOI:** 10.1080/23802359.2018.1462669

**Published:** 2018-06-12

**Authors:** Aaron W. Aunins, Donald Hamilton, Timothy L. King

**Affiliations:** a Government Natural Systems Analysts, Inc., under contract to the U.S. geological Survey, Kearneysville, WV, USA;; b National Park Service, Upper Delaware Scenic and Recreational River, Beach Lake, PA, USA;; c Aquatic Ecology Branch, USGS Leetown Science Center, United States Geological Survey, Kearneysville, WV, USA

**Keywords:** *Didymosphenia geminata*, didymo, stalk-forming diatom, mitogenomics

## Abstract

The complete mitogenome of the stalk-forming diatom *Didymosphenia geminata* collected from Mineral County, WV, USA was sequenced on the Ion Torrent PGM and Proton sequencers. The *D. geminata* mitogenome is 37,765 bp and encodes 35 protein coding genes, 25 tRNAs, and both large and small subunit ribosomal RNA genes. The *nad*11 gene is split into two domains as observed in *Phaeodactylum tricornutum*, and *D. geminata* also lacks the large repeat region found in the *P. tricornutum* mitogenome. Gene order and content within the *D. geminata* mitogenome is similar to the diatom *Berkeleya fennica*.

The freshwater stalk-forming diatom *Didymosphenia geminata* has been the focus of intense scientific investigations over the last decade because of the increasing frequency of large scale *D. geminata* ‘blooms’ in rivers and streams worldwide. The cause of these blooms remains poorly understood, though streams with low inorganic phosphorous and clear and cool water appear to be most at risk (Bergey and Spaulding 2015). A paucity of genetic data exists for *D. geminata*, but is necessary to better understand how *D. geminata* nuisance blooms form, as well as to better understand patterns of phylogeographic structure. Here, we describe the complete mitogenome of *D. geminata* to move forward genomic study of this nuisance species.

A 2 cm^3^ sample of *D. geminata* mat was obtained from the tailwaters of Jennings Randolph reservoir (39.4318°N –79.1152°W) in Mineral County, WV, March 2014. The sample is being stored at the U.S. Geological Survey Leetown Science Center, Kearneysville, WV, and 300 intact *D. geminata* detached from their stalks were isolated from this mat sample by mouth pipette for analysis. These cells were incubated in a lysozyme solution (20 mM Tris HCl pH 8, 2 mM sodium EDTA, 20 mg/mL lysozyme) for 10 minutes at 25 °C, and rinsed with 0.25X TE to reduce bacterial contamination in the subsequent Qiagen DNEasy DNA extraction. Libraries were prepared for single end sequencing on both the Ion Torrent PGM and Proton sequencers (Thermo Fisher Scientific, Frederick, MD, USA). *De novo* assembly of 14,018,492 sequence reads from the PGM and 39,227,730 sequence reads from the Proton in CLC Genomics Workbench (Qiagen, Frederick, MD, USA) resulted in four separate (but overlapping) contigs of mtDNA origin. A consensus of the overlapping contigs was generated and the reads were mapped back to this consensus resulting in a complete circular mapping mitogenome of 37,765 bp with an average coverage of 1218. Annotation of protein coding, ribosomal genes, and tRNAs was accomplished using the program MFANNOT (Beck and Lang 2010). A partitioned maximum likelihood phylogenetic tree with 1000 ultrafast bootstrap resamplings (Hoang et al. 2018) was created in the program IQ-Tree (Nguyen et al. 2015) using a MUSCLE alignment of 6817 amino acids (Edgar 2004) generated in the program TranslatorX (Abascal et al. 2010) of 27 shared protein coding genes (*atp*6, *atp*9, *cob*, *cox*1, *cox*2, *cox*3, *nad*11, *nad*1, *nad*2, *nad*3, *nad*4, *nad*4L, *nad*5, *nad*6, *nad*7, *nad*9, *rpl*14, *rpl*2, *rpl*5, *rps*10, *rps*12, *rps*13, *rps*14, *rps*19, *rps*3, *rps*4, *rps*8) from 7 other selected members of the class Bacillariophyceae with complete mitogenomes. The best partitioning scheme for the analysis was determined with PartitionFinder2 (Lanfear et al. 2017).

The *D. geminata* mitogenome (GB KX889125) codes for the small and large subunit rRNAs, 35 protein coding genes (*atp*6, 8, 9; *cob*; *cox*1, 2, 3; *nad*1-4, 4L, 5-7, 9,11a,11b; *rpl*2, 5, 6, 14, 16; *rps*2-4, 7, 8, 10-14, 19; *tat*C, *tat*A), and 25 tRNAs. The gene order was nearly identical to the mitogenome of the diatom *Berkeleya fennica* (An et al. 2016), with the exception of *D. geminata* having tRNA-Gln before *cox*1, and an extra tRNA-Ile after *nad*2. Start codons included ATG, GTG, and TTG, while stop codons included TAA, TAG, and TAA. Like within *Phaeodactylum tricornutum*, the *nad*11 gene is split into two domains in *D. geminata* (Oudot-Le Secq and Green 2011). However, *D. geminata* does not possess a large repeat region as observed in the *P. tricornutum* mitogenome. The maximum likelihood tree indicates that *D. geminata* forms a monophyletic clade with *Fistulifera solaris*, *Berkeleya fennica,* and *Phaeodactylum tricornutum*, but there is low bootstrap support for the relationships among *D. geminata*, *Berkeleya fennica*, and *Phaeodactylum tricornutum* (Figure 1).

**Figure 1.**
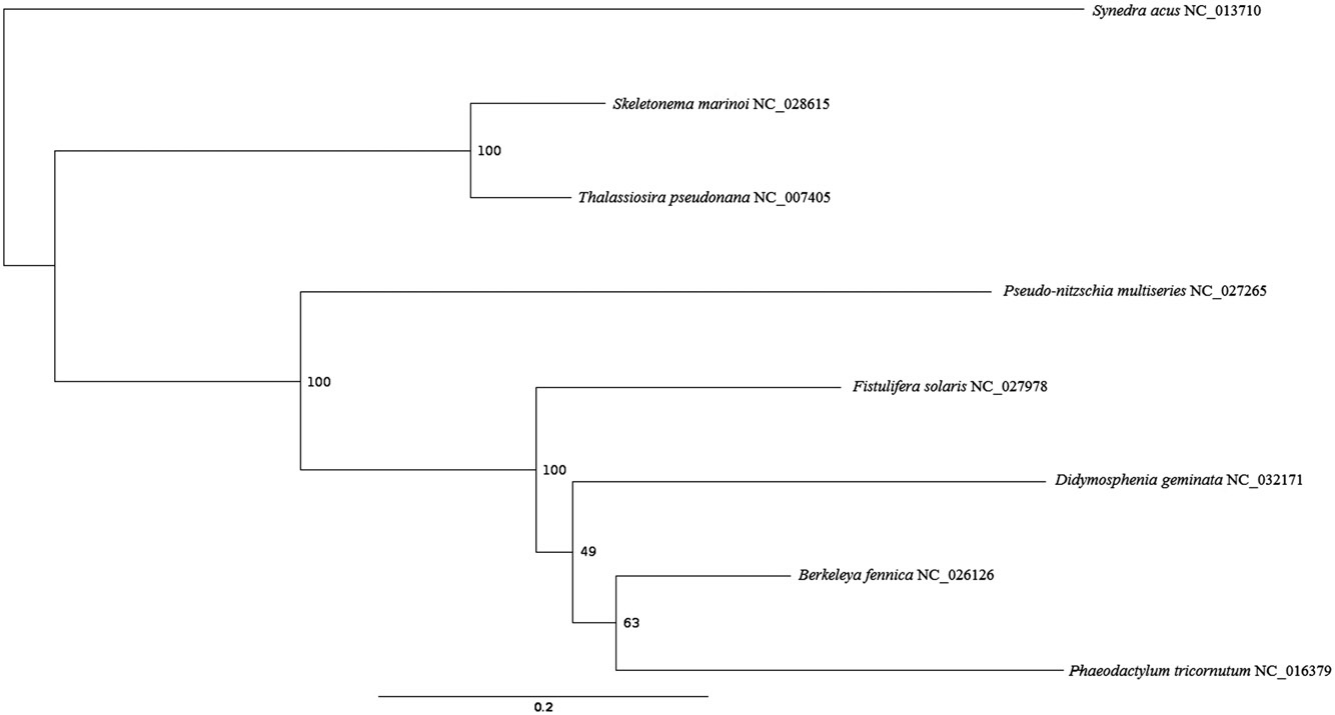
Partitioned maximum likelihood unrooted phylogenetic tree constructed in IQ-Tree (Nguyen et al. 2015) of 6817 amino acid positions with 1000 ultrafast bootstrap resamplings (Hoang et al. 2018) indicating the relationship of *Didymosphenia geminata* with seven other members of the class Bacillariophyceae based on 27 shared protein coding genes from the mitochondrial genome (atp6, atp9, cob, cox1, cox2, cox3, nad11, nad1, nad2, nad3, nad4, nad4L, nad5, nad6, nad7, nad9, rpl14, rpl2, rpl5, rps10, rps12, rps13, rps14, rps19, rps3, rps4, rps8). The partitioning scheme with the best corrected AIC score in PartitionFinder2 was used (Lanfear et al. 2017). GenBank accession numbers are next to the species names.
